# Assessing the Dissemination of Federal Risk Communication by News Media Outlets During Enteric Illness Outbreaks: Canadian Content Analysis

**DOI:** 10.2196/68724

**Published:** 2025-04-10

**Authors:** Hisba Shereefdeen, Lauren Elizabeth Grant, Vayshali Patel, Melissa MacKay, Andrew Papadopoulos, Leslie Cheng, Melissa Phypers, Jennifer Elizabeth McWhirter

**Affiliations:** 1 Department of Population Medicine, Ontario Veterinary College, University of Guelph, Population Medicine Building, Room 201, 50 Stone Rd E, Guelph, ON, N1G 2W1, Canada, 1 519-824-4120; 2 Outbreak Management Division, Centre for Foodborne, Environmental & Zoonotic Infectious Diseases, Public Health Agency of Canada, Guelph, ON, Canada

**Keywords:** risk communication, health communication, enteric illness, foodborne illness, zoonotic disease, media, content analysis, health belief model, public health, Canada

## Abstract

**Background:**

Effective dissemination of federal risk communication by news media during multijurisdictional enteric illness outbreaks can increase message reach to rapidly contain outbreaks, limit adverse outcomes, and promote informed decision-making by the public. However, dissemination of risk communication from the federal government by mass media has not been evaluated.

**Objective:**

This study aimed to describe and assess the dissemination of federal risk communication by news media outlets during multijurisdictional enteric illness outbreaks in Canada.

**Methods:**

A comprehensive systematic search of 2 databases, Canadian Newsstream and Canadian Business & Current Affairs, was run using search terms related to the source of enteric illnesses, general outbreak characteristics, and relevant enteric pathogen names to retrieve news media articles issued between 2014 and 2023, corresponding to 46 public health notices (PHNs) communicating information about multijurisdictional enteric illness outbreaks during the same period. A codebook comprised of 3 sections—general characteristics of the article, consistency and accuracy of information presented between PHNs and news media articles, and presence of health belief model constructs—was developed and applied to the dataset. Data were tabulated and visualized using RStudio (Posit).

**Results:**

News media communicated about almost all PHNs (44/46, 96%). News media commonly developed their own articles (320/528, 60.6%) to notify the public about an outbreak and its associated product recall (121/320, 37.8%), but rarely communicated about the conclusion of an outbreak (12/320, 3.8%). News media communicated most outbreak characteristics, such as the number of cases (237/319, 74.3%), but the number of deaths was communicated less than half the time (114/260, 43.8%). Benefit and barrier constructs of the health belief model were infrequently present (50/243, 20.6% and 15/243, 6.2%, respectively).

**Conclusions:**

Canadian news media disseminated information about most multijurisdictional enteric illness outbreaks. However, differences in coverage of multijurisdictional enteric illness outbreaks by news media were evident. Federal organizations can improve future risk communication of multijurisdictional enteric illness outbreaks by news media by maintaining and strengthening interorganizational connections and ensuring the information quality of PHNs as a key information source for news media.

## Introduction

Risk communication is a fundamental component of risk analysis that involves the exchange of information about risk and risk-related factors using a range of appropriate tools and strategies [1]. Effective risk communication is instrumental in promoting awareness [2], increasing behavior uptake [3], and mitigating adverse health events [4,5]. Food safety risk communication has 2 aims depending on the audience. For those in public and private sectors, it ensures a clear and transparent decision-making process to assess and manage food safety risks. For the public, it enhances awareness of relevant food safety risks and promotes health-protective behaviors and corrective actions for ill persons [6,7]. In our study, risk communication is defined as conveying information to increase awareness or promote behavior change to prevent exposure or manage illness during a multijurisdictional enteric illness outbreak.

Enteric illnesses are an important public health concern that impacts an estimated 4 million Canadians each year [8,9]. Enteric pathogens are transmitted via the fecal-oral route through consumption of contaminated food or water, animal contact, or person-to-person transmission [10-12]. Enteric illnesses are usually acute and self-limited but can cause serious illness or death, particularly in vulnerable populations [13-16]. In addition, enteric illnesses can result in chronic sequelae and morbidity following acute infection [17-20]. As a result, rapid and effective control of enteric illness outbreaks that limit potential widespread exposure to enteric pathogens and resultant adverse health outcomes and health care utilization is essential. One of the broadest and fastest tools that can be deployed once an outbreak is detected is risk communication to the public, and as such, effective risk communication is essential for controlling outbreaks and safeguarding human health [21-23].

In Canada, the response to multijurisdictional enteric illness outbreaks is guided by the Foodborne Illness Outbreak Response Protocol [24]. This protocol outlines the coordinated approach of cross-sectoral organizations, including the Public Health Agency of Canada (PHAC), the Canadian Food Inspection Agency (CFIA), Health Canada, and provincial or territorial organizations. When an enteric illness outbreak occurs in 2 or more provinces or territories or between Canada and another country (multijurisdictional), PHAC manages the outbreak response to ensure rapid and coordinated action across multiple jurisdictions [24]. If deemed necessary, PHAC will issue web-based public health notices (PHNs) and social media posts to communicate outbreak information and behavioral recommendations [24]. This information is intended to be used by health organizations, news media, and the public as a central source of factual, timely information.

News media are not required to convey outbreak information; however, their ability to relay information and influence health decision-making make them an important communication pathway during an outbreak [25-27]. News media use multiple communication channels to reach their target audiences including traditional (eg, television and print media) and online formats (eg, web, social media, and podcasts). These platforms are a popular source of health information among Canadians, with 78% relying on the internet to access health information during the COVID-19 pandemic [28]. Given that 95% of Canadians are internet users, online formats have the broadest potential reach. On average, Canadian adults spend over 1 hour reading press media online each day [29]. Despite the digitalization of print publications, print newspapers remain the most trusted news source for Canadians [30]. Both print and digital newspapers maintain high readership across generations, with 83% of Canadian millennials, 83% of Gen X adults, and 78% of boomers engaging with these formats [31]. As intermediaries between public health agencies and the public, news media have the potential to effectively shape how risk information is framed and received. However, the coverage of information may be influenced by factors beyond public health priorities, such as agenda-setting priorities, where news media may highlight or downplay certain information to promote or limit the public’s understanding [32-34].

News media are valuable information sources with broad public reach that can disseminate PHAC’s public communication during multijurisdictional enteric illness outbreaks to ensure timely delivery of information to at-risk populations. Given their potential reach and impact, it is imperative to optimize this communication pathway to maximize the number of Canadians receiving information during an outbreak. However, a formal assessment of how and the extent to which news media disseminate federal risk communication in this context has not been conducted. This information is needed to inform how public communication during enteric illness outbreaks can be improved to increase access to information and support adoption of protective behaviors, access to appropriate treatment options, and ultimately limit adverse population health outcomes. In this study, dissemination is assessed by the frequency of communication, determined by the number of news media articles, and the inclusion of information from PHNs in news media articles. The aim of this study is to assess the communication pathway between PHAC and news media by describing how, and the extent to which, Canadian news media disseminate federal risk communication during multijurisdictional enteric illness outbreaks.

## Methods

### Data Collection

In consultation with the research team and a Research Librarian from the University of Guelph, search strings in English and French were developed using a combination of general enteric illness outbreak terms and specific pathogens that have caused multijurisdictional enteric illness outbreaks in Canada:

1. (food OR waterborne OR zoono* OR case? OR illness* OR contamina* OR outbreak? OR recall* OR toxic* OR advisor* OR alert* OR sick* OR disease*) AND (norovirus OR salmonella OR cyclospora OR hepatitis OR hep OR listeria OR listeriosis OR vibrio OR Ecoli OR “E.coli” OR “E coli” OR “E. coli” OR “Escherichia coli” OR Pseudomonas OR botulism).
2. (aliment OR “maladies d’origine hydrique” OR “zoonotiques” OR cas OR maladie, OR contaminent OR éclosion OR rappel OR toxique OR avis OR alerte OR malade) AND (norovirus OR salmonella OR Cyclospora OR hépatite OR listeria OR listériose OR vibrio OR Ecoli OR “E.coli” OR “E coli” OR “E. coli” OR “Escherichia coli” OR pseudomonas OR botulisme).

Search strings were applied to the Canadian Newsstream and Canadian Business & Current Affairs databases, accessible through the University of Guelph, on November 12, 2023. Data were excluded if they did not meet the eligibility criteria, which were as follows: the news article must (1) communicate about a multijurisdictional enteric illness outbreak (eg, original outbreak notice, updates about outbreaks, or food recall notice) in Canada; (2) be published between January 1, 2014, and November 12, 2023, referencing a multijurisdictional enteric illness outbreak for which there is an available PHN on the Government of Canada website [35]; (3) be published by at least 1 Canadian news media outlet; (4) be written in English or French; and (5) have been originally communicated in written format. A start date of 2014 was selected as, at the time of writing, PHNs published before 2014 were archived and not publicly accessible. These eligibility criteria and search terms were also applied to a targeted Google search of randomly selected news media outlets (Multimedia Appendix 1). Retrieved results were exported and uploaded to Covidence (Veritas Health Innovation) [36], where articles were deduplicated and underwent title and abstract and full-text screening by 2 researchers (HS and VP).

At the time of data collection, a unique ID, a numerical identifier corresponding to the relevant PHN (Multimedia Appendix 2), and the geographical scope of the news media outlet were assigned. Local and regional news media were distinguished by their coverage areas, with local outlets providing information to specific neighborhoods or towns, while regional outlets encompassed broader regions, such as provinces or territories.

### Content Analysis

The content analysis used the health belief model (HBM) [37] as a theoretical framework and followed a previously described established methodology for qualitative content analysis [38]. The codebook (Multimedia Appendix 3) was comprised of 3 sections. Section 1 collected information about the general characteristics of the news article, including the language of communication, type of article, and intention of the article.

Section 2 assessed the inclusion of outbreak information in news media articles to evaluate the accuracy and consistency of information communicated between PHNs and news media articles (recall of item, location of the outbreak, morbidity and mortality, health protective behaviors, and symptoms). In our study, consistency refers to the regular reporting of outbreak characteristics. This includes always reporting the number of illnesses, or explicitly stating the source when this information is available. Accuracy, which is conceptualized as the reporting of the same information indicated in PHAC’s PHNs, includes indicating the correct number of hospitalizations. Consistency and accuracy were determined by tracing the posting date of news media articles and comparing the information with that presented in PHNs of the same period.

Section 3 included constructs from the HBM, a theoretical framework that posits an individual’s motivation to implement a behavior is influenced by their perception of the severity and susceptibility of a given outcome [37,39]. These constructs include (1) cues to action, which describe any stimuli that promotes the implementation of a recommended behavior, (2) self-efficacy, which refers to the level of confidence an individual has in performing the recommended behavior, (3) perceived susceptibility, which refers to an individuals’ subjective perception of the risk of acquiring an illness or disease, (4) perceived severity, which refers to an individuals’ perception of the seriousness of the illness or disease, (5) perceived benefits, which refer to an individual’s perception of the effectiveness of the behaviors intended to reduce the threat of an illness or disease, and (6) perceived barriers, which refer to the individual’s perception of the obstacles to performing the recommended behaviors. In this study, self-efficacy was conceptualized as communicating about, or encouraging the public, to perform the recommended behaviors outlined in a news media article.

### Data Analysis

A 10% (77/763) sample of news media articles was independently coded by 2 researchers (HS and VP), and the Cohen κ statistic was calculated using the *irr* package in R Studio v.4.2.3 (Posit) to assess interrater reliability. A value of 10% was chosen by the research teams to ensure that a sufficient subsample was analyzed. The statistical analysis indicated almost perfect agreement between the 2 coders (κ=0.9) [40]. The 2 researchers resolved disagreements in coding, and no significant changes were made to the codebook. The remaining articles were evenly split and coded by HS (n=343) and VP (n=343). All coding was performed using Microsoft Excel.

Total and stratified descriptive statistics were calculated. We examined differences in general characteristics of unique original news media articles by geographic scope (national [n=97], regional [n=123], and local [n=100]) and by source of outbreak (foodborne [n=286], zoonotic [n=7], and unknown [n=27]). In addition, we examined differences in the inclusion of HBM constructs in original news media articles providing at least 1 behavioral recommendation (n=243) by geographic scope (national [n=68], regional [n=94], and local [n=81]) and by source of outbreak (foodborne [n=228], zoonotic [n=4], and unknown [n=11]). Chi-square tests were used to test for differences in communication variables. A significance level of .05, a conventionally accepted threshold, was used to determine statistical significance. All analyses were conducted and visualized using RStudio base packages, *tidyr* [41] and *ggplot2* [42].

### Ethical Considerations

Ethics approval was not required. This study used publicly available data from news media articles indexed in the Canadian Newsstream and Canadian Business & Current Affairs databases.

## Results

### Dissemination of PHN Information by News Media

#### Dataset Creation

**Figure 1.**
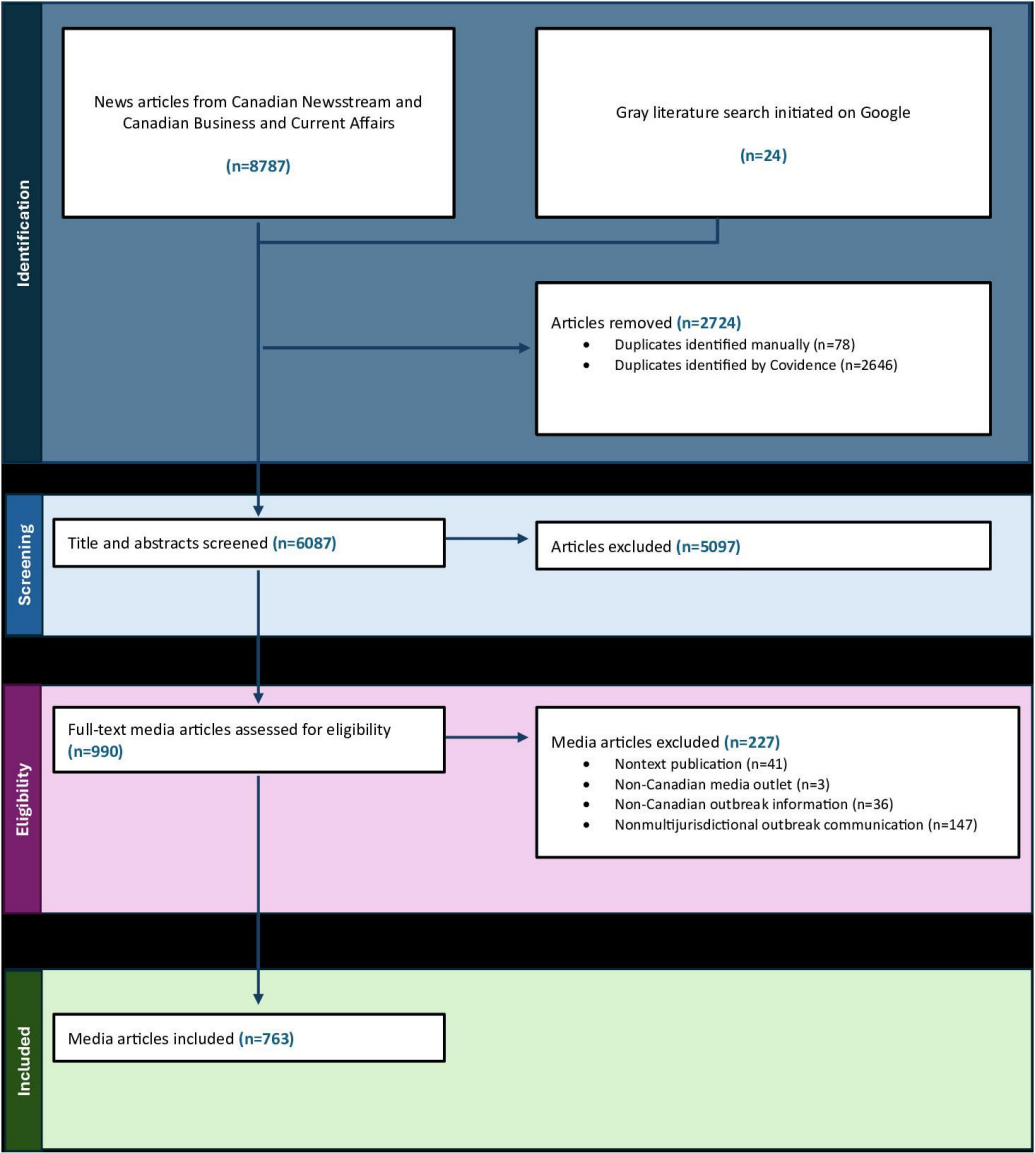
PRISMA (Preferred Reporting Items for Systematic reviews and Meta-Analyses) diagram depicting the systematic retrieval of news media articles from databases and a targeted Google search.

Citation searches identified 8787 news media articles from news databases (Figure 1). After removal of duplicate citations (n=2724), 6063 database articles and 24 gray literature articles underwent title and abstract screening. After screening, 5097 news media articles were excluded. Full-text news media articles (n=990) were then assessed for their eligibility, and 227 articles were excluded because they did not communicate about a multijurisdictional enteric illness outbreak (n=147), were nontext publications (n=41), did not communicate about Canadian outbreaks (n=36), or were issued by a non-Canadian news media outlet (n=3). This process yielded 763 unique articles related to 46 PHNs.

#### Extent of Dissemination per PHN

**Figure 2.**
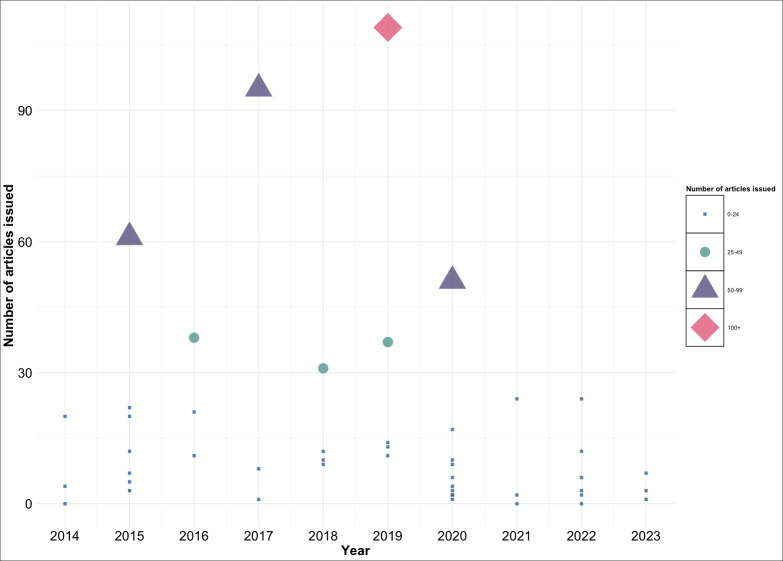
Bubble chart visualizing the number of news media articles issued per public health notice. Each bubble represents 1 public health notice. The size and color of the bubble correlates to the number of posts issued for the public health notice.

News media communicated about almost all PHNs (44/46, 96%) by issuing at least 1 article per PHN (Figure 2). On average, news media issued 16.6 (SD 22.7) articles (0‐109 articles) per PHN. The most disseminated PHNs by news media were the 2017‐2019 *Salmonella* outbreaks involving frozen raw breaded chicken products (109/763, 14.3%) and the 2017 *Escherichia coli* (*E. coli*) O121 PHN involving flour (95/763, 12.5%). The remaining PHNs were disseminated 0‐65 times each, with the majority of PHNs being disseminated up to 10 times each (Multimedia Appendix 4).

### Content Analysis

#### Dataset Creation

Content analysis of original news media articles further excluded articles that shared official government and industry statements verbatim (n=208; PHNs [n=127]; food recall notices [n=73]; industry statements [n=6]; other Government of Canada statements [n=2]), yielding 555 articles. In addition, duplicates were removed (n=235), yielding 320 unique original news media articles for content analysis. Duplicates included the same news media article published by multiple outlets or in both English and French. Where relevant, the denominator representing the number of articles was further adjusted to support valid interpretations and is indicated in the text.

#### Communication Approaches

Original news media articles were most often issued by regional news media (123/320, 38.4%), followed by local news media (100/320, 31.3%), and national news media (97/320, 30.3%) (Table 1). Considering both original news media articles (n=320) and resharing of verbatim statements (n=208), 60.6% (320/528) of articles were original news media articles and 24.1% (127/528) shared PHNs verbatim. For articles published about a PHN with an associated food recall (n=378), 19.3% (73/378) shared food recall notices verbatim. To assess language distribution, we considered both original news media articles and duplicates to avoid excluding French communications (n=555). English was the predominant language used (533/555, 96%) compared with French (22/555, 4%). The language of news media articles significantly differed by the scope of news media coverage (Multimedia Appendix 5): local news media produced only 1 article (1/239, 0.4%) in French and regional news media only communicated in English (188/188, 100%; *P*<.001).

Most original news media articles were regarding foodborne outbreaks (286/320, 89.3%) (Table 2). Other news media articles communicated about zoonotic outbreaks (7/320, 2.2%) and outbreaks where the source was unknown (27/320, 8.4%). Original news media articles often notified the public of an ongoing food recall (121/320, 37.8%). Others were intended to provide an initial notice about an outbreak (107/320, 33.4%). News media articles notifying the public about the end of an outbreak were infrequent (12/320, 3.8%). For original news media articles published regarding an outbreak with an associated recall (n=204), news media consistently mentioned the ongoing recall of products (194/204, 95.1%).

**Table 1.** Descriptive statistics of variables assessing the communication methods by news media.

Variable and variable level		Value, n (%)
Geographic scope of news media article (n=320)		
	Regional	123 (38.4)
	Local	100 (31.3)
	National	97 (30.3)
Type of news media article (n=528; 320+208 verbatim articles)		
	Original article by news media outlet	320 (60.6)
	Shares PHN^a^ verbatim	127 (24.1)
	Shares CFIA’s^b^ food recall notices verbatim (n=378 articles for PHNs with an associated recall)	73 (19.3)
	Shares official industry communication verbatim (eg, Safety Notice by Producer)	6^c^
	Shares other official communication verbatim (eg, statement by the Chief Public Health Officer obf Canada)	2^c^
Language of news media article (n=555; 320+235 duplicates)		
	English	533 (96)
	French	22 (4)

a PHN: public health notice.

b CFIA: Canadian Food Inspection Agency.

c Percentage value (%) not applicable.

**Table 2.** Descriptive statistics of variables assessing the general characteristics of news media articles.

Variable and variable level		Value, n (%)
Distribution of news media articles by source type (n=320)		
	Foodborne	286 (89.3)
	Unknown	27 (8.4)
	Zoonotic	7 (2.2)
Intention of news media article (n=320)		
	Food recall notice	121 (37.8)
	Notice of outbreak or advisory	107 (33.4)
	Update of ongoing outbreak	30 (9.4)
	Unclear	30 (9.4)
	Reminder or best practice	19 (5.9)
	End of outbreak or advisory	12 (3.8)
	Other	1 (0.3)
Indication of item recall (n=204; 378 articles for PHNs^a^ with an associated recall – 159 shared articles – 15 articles where recall issued after article was published)		
	Yes	194 (95.1)
	No	10 (4.9)

a PHN: public health notice.

#### Inclusion of Outbreak Characteristics and Preventive Behavioral Recommendations

Considering news media articles where the associated PHN had relevant information regarding outbreak characteristics and behavioral recommendations, news media articles commonly included the number of cases (237/319, 74.3%), and to a lesser extent, the location of the outbreak (181/316, 57.3%), number of hospitalizations (144/277, 52%), and number of deaths (114/260, 43.8%) (Multimedia Appendix 5). Local news media less frequently communicated about several outbreak characteristics, including the location of the outbreak (48/100, 48%; *P*=.04), number of hospitalizations (33/94, 35%; *P*<.001), and number of deaths (24/77, 31%; *P*=.03) compared with regional and national news media.

News media articles often included preventive behavior information (243/320, 75.9%) and symptoms of illness (201/320, 62.8%) (Table 3). For news media articles that included at least 1 behavioral recommendation (n=243), behavioral recommendations if an individual becomes ill (eg, visiting the doctor if you become ill) were not commonly communicated (62/243, 25.5%). Local news media more frequently provided behavioral recommendations for what to do if an individual becomes ill (29/81, 36%; *P*=.01) (Multimedia Appendix 5).

**Table 3.** Descriptive statistics of variables assessing how news media promotes behavioral uptake.

Variable and variable level		Value, n (%)
Indication of symptoms (n=320)		
	Yes	201 (62.8)
	No	119 (37.2)
Provision of behavioral recommendations to prevent illness (n=320)		
	Yes	243 (75.9)
	No	77 (24.1)
Provision of behavioral recommendations if individual becomes ill (n=243)		
	Yes	62 (25.5)
	No	181 (74.6)

#### Inclusion of HBM Constructs in News Media Articles With Behavioral Recommendations

Considering news media articles with at least 1 behavioral recommendation provided (n=243), cues to action (202/243, 83.1%) and self-efficacy constructs (191/243, 78.6%) were often present. National news media articles less frequently included the self-efficacy construct (44/68, 65%) compared with local (68/81, 84%) and regional news media (79/94, 84%; *P*=.004). Likewise, news media articles about zoonotic outbreaks never contained the self-efficacy construct, whereas articles about foodborne outbreaks frequently included this construct (187/228, 82%; *P*<.001). News media articles often mentioned information relevant to the susceptibility (190/243, 78.2%) and severity of illness (161/243, 66.3%). However, they less commonly included information pertaining to the benefits of implementing the recommended behaviors (50/243, 20.6%), and seldom addressed the potential barriers an individual may encounter (15/243, 6.2%).

## Discussion

### Principal Findings

Effective communication during enteric illness outbreaks is important for disease control. Coordination of communication efforts and repetition of information across sources are important for increasing the public’s receptiveness to the message. To describe the dissemination of federal risk communication during multijurisdictional enteric illness outbreaks by news media, we performed a content analysis of news media articles issued by local, regional, and national Canadian outlets over a 9-year period. The media plays an important role in risk management as an arms-length source of information about government decisions and responses to a health event by contributing to the public’s awareness of hazards and influencing their behavior [43]. The ability to effectively communicate timely, accurate, and clear information to the media is a key responsibility of public health, especially during an emergency [44]. An evaluation of news media communication during multijurisdictional enteric illness outbreaks provides a snapshot of how news media disseminate federal risk communication during an outbreak. It also identifies key areas of opportunity to improve this communication pathway.

### Differences in News Media Dissemination of PHNs and the Importance of Coordinated Risk Communication

Effective dissemination of critical health information by news media can enhance message reach and visibility to the public and promote adherence to recommended behaviors. During the COVID-19 pandemic, Canadians frequently sought information from online sources that were not government or public health web pages [45]. They also perceived news media to be equally credible to national government and public health websites [45], indicating that news media are trusted sources that can effectively promote the uptake of protective behaviors.

Our results indicate that local, regional, and national news media communicated about almost all PHNs at least once, primarily in English. While the scope of media sources in our study was predominantly regional, outbreak communication by national sources included dissemination by wire services, highlighting the wide reach of information to diverse audiences across the country. As expected, most news media articles were about foodborne outbreaks. However, dissemination per PHN varied, with 2 PHNs being disseminated to a greater extent than others. The PHN with the highest media coverage, with 109 news media articles, occurred between 2017 and 2019 and was associated with raw chicken and frozen raw breaded chicken products [46]. The second most disseminated PHN, with 95 news media articles, was the 2017 *E. coli* O121 PHN associated with flour [47]. This outbreak prompted a significant nationwide recall directed at consumers, restaurants, and retailers. The coverage of these high-risk outbreaks is expected and in alignment with other studies showing that news media coverage increases during significant public health events, such as large food recalls [48]. In addition, the 2017‐2019 PHN associated with frozen raw breaded chicken products covered 18 individual outbreaks; however, they were treated as a single multijurisdictional outbreak due to the issuance of only 1 PHN. This may have overlooked distinctions in the extent of dissemination between individual outbreaks.

Ensuring that all multijurisdictional enteric outbreaks are disseminated is important for increasing the reach and visibility of protective information. A qualitative evaluation of the Centers for Disease Control and Prevention’s risk communication methods during multistate foodborne outbreaks revealed that consumers want to be notified about all outbreaks, regardless of their direct relevance [49]. Journalists have identified information dissemination as their primary role during food incidents [50], underscoring the importance of close collaboration between public health officials and news media to ensure regular and consistent communication of information to the public.

### Importance of Consistent, Accurate, and Timely Communication in Dissemination of PHNs by News Media

When faced with uncertainty, individuals seek information from various sources, including news media [51], highlighting the need for accurate and consistent information. A key component of effective risk communication is the communication of accurate and consistent information by stakeholders [52], as this enables the public to understand the risks and implications of the outbreak, facilitating informed decision-making and increasing the uptake of protective behaviors [53].

Although traditional media sources generally overreport death tolls [54,55], our study found a significant difference in reporting across local, regional, and national news media, with a general trend toward underreporting case fatality numbers. While sensationalized reporting often contributes to public fear and erosion of trust [56,57], Zhang et al [58] found an increased implementation of protective actions during the H1N1 flu outbreaks when individuals were exposed to media coverage that incited fear. Nevertheless, reporting of death tolls is important for providing the public with information necessary to make informed health decisions. Inaccurate reporting can heighten public fear [51] and undermine trust in risk communication [59]. In some cases, downplaying health risks resulted in reduced public urgency to implement health protective behaviors [60].

News media often communicated about ongoing food recalls associated with an enteric illness outbreak, with some news media articles directly sharing food recall notices shared by CFIA. Often, CFIA’s food recall notices directed readers to visit PHNs to learn more about the outbreak. This enables the public to avoid contaminated food and protect themselves against illness, an important goal during outbreaks. This study noted frequent communication at the onset of multijurisdictional enteric illness outbreaks; however, communication about the conclusion of an outbreak was less common. This may impact the public’s access to timely information. Outbreaks often evolve rapidly, posing potential challenges for news media to access up-to-date information. However, timely risk communication is important for ensuring that the public is aware of how outbreaks evolve over time [61].

### Integration of HBM Constructs When Communicating About Multijurisdictional Enteric Illness Outbreaks

News media can importantly influence the public’s perception of the outbreak [43]. Risk perception is influenced by threat severity; that is, how susceptible an individual feels to the threat or hazard, and the perceived efficacy of protecting themselves [53]. Models, such as the HBM, provide guidance for health message development that can influence risk perception and encourage the uptake of risk-protective behaviors [62]. To assess how news media disseminate information that helps the public to safeguard their health, we examined the inclusion of HBM constructs. These constructs provide an assessment of whether the public can act on the information being communicated, aligning with the core objective of food safety risk communication, which promotes informed decision-making and uptake of protective behaviors [7].

In this study, news media consistently incorporated the cues to action construct when disseminating PHAC’s public communication during multijurisdictional enteric illness outbreaks. Cues to action can increase behavioral acceptance [63], and news media regularly provided recommendations during an outbreak and after its conclusion. These included advising the public to check their shelves for the presence of recalled items. In addition, news media frequently mentioned symptoms associated with the illness. While news media regularly provided cues to action such as behavioral recommendations for individuals to prevent the onset of illness, our study noted the infrequent provision of behavioral recommendations for ill persons (eg, encouraging ill individuals to visit the doctor) by national news media and when communicating about zoonotic outbreaks. Cues to action are important for individuals to develop readiness to act in response to a perceived threat [64] and should be provided to enhance risk perception.

Self-efficacy is particularly crucial in food safety communication, as this construct promotes safe food handling intentions [65]. In this study, self-efficacy was conceptualized as communication about or encouraging the public to perform the recommended behaviors using the health protective behaviors outlined in news media articles. For instance, when directing consumers to wash lettuce to prevent *E. coli* contamination, news media often provided specific recommendations, including the length of time and how each leaf should be washed. While the self-efficacy construct was generally present in news media articles, our study noted that articles by national news media conveyed this construct less frequently compared to local and regional sources. In addition, reporting of this construct was absent in articles regarding zoonotic outbreaks. It is important that news media articles integrate the self-efficacy construct, as this construct influences the public’s confidence in implementing the recommended behaviors [66].

Susceptibility and severity constructs were also frequently present in news media articles. These constructs directly influence risk perception and are crucial factors driving the uptake of behavioral recommendations [67]. News media often communicated about the susceptibility of high-risk groups, such as pregnant women, for contracting the illness. Similarly, news media provided information about the fatality of some enteric pathogens, encouraging individuals to consider their vulnerability to the illness and the seriousness and consequences of the outbreak.

Benefits and barrier constructs, observed less frequently in news media articles, are thought to be the strongest predictors of behavioral change [68]. News media frequently reported the susceptibility and severity of illness, while underreporting the benefits and barriers an individual may face when performing the recommended behaviors. News media is more inclined to sensationalize risk information by overemphasizing risks and negative consequences [69], impeding risk perception [55,70]. In food safety, it is vital to communicate about the risks, benefits, and barriers as this facilitates informed food choices [7]. Although this study did not assess federal risk communication materials, it is important to note that HBM constructs may not be present in official federal communication products such as PHNs. It is reasonable to expect that news media will reflect the information presented in PHNs. In addition, other factors, such as agenda-setting priorities, may influence what and how information is presented to the audience. For instance, news media may emphasize the severity of an outbreak as opposed to the benefits of implementing a protective behavior, as this can lead to increased audience engagement [71]. However, federal partners and news media may benefit from integrating all HBM constructs in future risk communication efforts by highlighting the ease of implementing recommended behaviours, emphasizing their positive impact on health, and addressing common concerns that may act as barriers to promoting health behavior.

When constructing risk communication messages, using the HBM constructs in combination may be effective in promoting behavior change [62,68,72,73]. For instance, risk communication could include stimuli to cue behavior change, instill confidence to adopt behavioral recommendations, and provide information about the susceptibility and severity of illness, and benefits and barriers an individual may encounter when performing the recommended behavior.

### Limitations

First, the data collection process involved running search strings on 2 databases accessed through the University of Guelph. While these databases cover a range of news outlets, not all Canadian news media may be indexed. Similarly, the gray literature search was limited to a subset of Canadian media outlets; this may have not captured all news media articles on multijurisdictional enteric illness outbreaks. Therefore, dissemination may be higher than observed in this study. In addition, the search strings used in this study, while comprehensive, may not have captured all news media articles related to multijurisdictional enteric illness outbreaks.

Second, this study assessed the presence of HBM constructs in articles issued by news media. However, this information may not be reflected in PHAC’s communication products and, as a result, may be absent from news media articles. Comparing these constructs between the 2 sources was outside this study’s scope. Future research should build on our findings and seek to better understand the inclusion of HBM constructs in PHNs and subsequent news media articles.

### Conclusions

This study explored the dissemination of federal risk communication by news media during multijurisdictional enteric illness outbreaks. Our findings suggest news media disseminated information on almost all multijurisdictional enteric illness outbreaks; however, there are opportunities for improving how federal risk communication is disseminated by news media. Federal organizations can improve future risk communication of multijurisdictional enteric illness outbreaks by news media by maintaining and strengthening interorganizational connections and ensuring the information quality of PHNs as a key information source for news media. Optimized communication can help to ensure the public has the information necessary to implement protective behaviors.

## Supplementary material

10.2196/68724 Multimedia Appendix 1 Results of gray literature searches of 4 Canadian news media outlets.

10.2196/68724 Multimedia Appendix 2 Multijurisdictional enteric illness outbreaks in Canada from 2014 to 2023.

10.2196/68724 Multimedia Appendix 3 Codebook used for media content analysis.

10.2196/68724 Multimedia Appendix 4 Distribution of multijurisdictional enteric illness outbreaks by number of news media articles issued.

10.2196/68724 Multimedia Appendix 5 Descriptive statistics of communication variables stratified by scope of news media coverage or outbreak source.

## Abbreviations

CFIA: Canadian Food Inspection Agency
HBM: health belief model
PHAC: Public Health Agency of Canada
PHN: public health notice
