# Supplementary material for: Assessing the Dissemination of Federal Risk Communication by News Media Outlets During Enteric Illness Outbreaks: Canadian Content Analysis
Source: JMIR Public Health Surveill. 2025 Apr 10;11:e68724. doi: 10.2196/68724 (PMC12005601; doi:10.2196/68724)
Supplement: Multimedia Appendix 1 [file publichealth-v11-e68724-s001.docx]

Grey literature search of four Canadian media outlets

**Media outlet 1: The Globe and Mail**

|  | Reviewer 1 (HS) | Reviewer 2 (VP) | Reviewer 1 (HS) | Reviewer 2 (VP) |  |
| --- | --- | --- | --- | --- | --- |
| **Search String** | **Date of Search** | **Date of Search** | **Number of Results** | **Number of Results** | **Links to Articles** |
| (food OR waterborne OR zoono* OR case? OR illness* OR contamina* OR outbreak? OR recall* OR toxic* OR advisor* OR alert* OR sick* OR disease*) AND (norovirus OR salmonella OR cyclospora OR hepatitis OR hep OR listeria OR listeriosis  OR vibrio OR Ecoli OR "E.coli" OR “E coli” OR “E. coli” OR “Escherichia coli” OR Pseudomonas OR botulism) | Jan 3, 2024 | Jan 10, 2024 | 59 | 113 | <https://www.theglobeandmail.com/news/national/robin-hood-all-purpose-flour-recalled-for-possible-e-coli-contamination/article34457879/>  <https://www.theglobeandmail.com/life/health-and-fitness/article-raw-chicken-even-frozen-breaded-kind-requires-safe-handling/>  <https://www.theglobeandmail.com/canada/article-romaine-lettuce-particularly-susceptible-to-e-coli-outbreaks-2/>  <https://www.theglobeandmail.com/canada/article-canadas-public-health-agency-says-romaine-lettuce-is-safe-to-eat/>  <https://www.theglobeandmail.com/life/health-and-fitness/article-is-it-safe-to-eat-romaine-lettuce-or-kale/> |
| (aliment OR "maladies d’origine hydrique" OR "zoonotiques" OR cas OR maladie OR contaminent OR éclosion OR rappel OR toxique OR avis OR alerte OR malade) AND (norovirus OR salmonella OR Cyclospora) | Jan 10, 2024 | Jan 10, 2024 | 0 | 0 |  |
| (aliment OR "maladies d’origine hydrique" OR "zoonotiques" OR cas OR maladie OR contaminent OR éclosion OR rappel OR toxique OR avis OR alerte OR malade) AND (hépatite OU listeria OU listériose) | Jan 10, 2024 | Jan 10, 2024 | 0 | 0 |  |
| (aliment OR "maladies d’origine  hydrique" OR "zoonotiques" OR cas OR maladie OR contaminent OR éclosion OR rappel OR toxique OR avis OR alerte OR malade) AND (vibrio OR Ecoli OR “E.coli”) | Jan 10, 2024 | Jan 10, 2024 | 0 | 0 |  |
| (aliment OR "maladies d’origine  hydrique" OR "zoonotiques" OR cas OR maladie OR contaminent OR éclosion OR rappel OR toxique OR avis OR alerte OR malade) AND (“E coli” OR “E. coli” OR “Escherichia coli”) | Jan 10, 2024 | Jan 10, 2024 | 0 | 0 |  |
| (aliment OR "maladies d’origine  hydrique" OR "zoonotiques" OR cas OR maladie OR contaminent OR éclosion OR rappel OR toxique OR avis OR alerte OR malade) AND (pseudomonas OR botulisme) | Jan 10, 2024 | Jan 10, 2024 | 0 | 0 |  |

**Media outlet 2: Toronto Star**

|  | Reviewer 1 (HS) | Reviewer 2 (VP) | Reviewer 1 (HS) | Reviewer 2 (VP) |  |
| --- | --- | --- | --- | --- | --- |
| **Search String** | **Date of Search** | **Date of Search** | **Number of Results** | **Number of Results** | **Links to Articles** |
| (food OR waterborne OR zoono* OR case? OR illness* OR contamina* OR outbreak? OR recall* OR toxic* OR advisor* OR alert* OR sick* OR disease*) AND (norovirus OR salmonella OR cyclospora OR hepatitis OR hep OR listeria OR listeriosis  OR vibrio OR Ecoli OR "E.coli" OR “E coli” OR “E. coli” OR “Escherichia coli” OR Pseudomonas OR botulism) | Jan 3, 2024 | Jan 11, 2024 | 300 | 313 | <https://www.thestar.com/news/gta/norovirus-outbreak-prompts-oyster-recall-by-b-c-company/article_ab9280cb-6f28-5ae1-9ac3-06e5f1589109.html>  <https://www.thestar.com/news/canada/union-bay-seafood-brand-pacific-oysters-recalled-due-to-norovirus-risk/article_dfb6a13b-6336-5544-818e-54b1bb6536db.html>  <https://www.thestar.com/life/health-wellness/3-people-from-canada-sickened-in-botulism-outbreak-in-bordeaux-france/article_b9c54eac-b2c4-57ba-ad79-65fd50375c92.html>  <https://www.thestar.com/news/canada/robin-hood-all-purpose-flour-recalled-for-possible-e-coli-contamination/article_d9423fd4-cc0c-57ad-b691-3cba5c50fe16.html>  <https://www.thestar.com/news/canada/loblaw-recalls-no-name-chicken-nuggets-due-to-possible-salmonella-contamination/article_b06d7851-c8bc-5b17-b8ab-7a271f3db681.html>  <https://www.thestar.com/news/world/e-coli-outbreak-warning-in-u-s-expands-to-all-romaine-lettuce/article_3c8f20dd-bd07-5518-858a-736707ae54e6.html>  <https://www.thestar.com/news/canada/public-health-notice-warns-of-norovirus-outbreak-linked-to-b-c-oysters/article_dacaaa6e-c612-5ddf-887b-327f5d0b4ce6.html>  https://www.thestar.com/news/canada/public-health-officials-investigating-cause-of-salmonella-outbreak-in-western-canada/article_955ecb5e-afc2-5381-a736-b0f45c8fb4c1.html  <https://www.thestar.com/news/canada/u-s-canadian-regulators-tie-hepatitis-cases-to-organic-strawberries/article_1b635418-f72d-560c-b58d-08812706a3e7.html>  <https://www.thestar.com/news/canada/recalled-costco-frozen-berries-linked-to-13-cases-of-hepatitis-a/article_4589cecf-f7f5-513f-bcce-2929f4dcab8d.html>  <https://www.thestar.com/news/canada/nature-s-touch-frozen-berry-mix-sold-at-costco-recalled-over-hepatitis-a-concerns/article_ed1f941b-44f5-5ae7-9e52-5bc022897c2c.html>  <https://www.thestar.com/news/canada/more-yellow-onions-sold-in-multiple-provinces-recalled-due-to-possible-salmonella/article_9b026477-eff1-504a-bcba-2ef661c9acb0.html>  <https://www.thestar.com/vancouver/centre-for-disease-control-says-norovirus-outbreak-linked-to-oysters-has-ended/article_7c359031-f743-58e3-b90d-c051589db596.html> |
| (aliment OR "maladies d’origine hydrique" OR "zoonotiques" OR cas OR maladie OR contaminent OR éclosion OR rappel OR toxique OR avis OR alerte OR malade) AND (norovirus OR salmonella OR Cyclospora) | Jan 10, 2024 | Jan 10, 2024 | 0 | 0 |  |
| (aliment OR "maladies d’origine hydrique" OR "zoonotiques" OR cas OR maladie OR contaminent OR éclosion OR rappel OR toxique OR avis OR alerte OR malade) AND (hépatite OU listeria OU listériose) | Jan 10, 2024 | Jan 10, 2024 | 0 | 0 |  |
| (aliment OR "maladies d’origine  hydrique" OR "zoonotiques" OR cas OR maladie OR contaminent OR éclosion OR rappel OR toxique OR avis OR alerte OR malade) AND (vibrio OR Ecoli OR “E.coli”) | Jan 10, 2024 | Jan 10, 2024 | 0 | 0 |  |
| (aliment OR "maladies d’origine  hydrique" OR "zoonotiques" OR cas OR maladie OR contaminent OR éclosion OR rappel OR toxique OR avis OR alerte OR malade) AND (“E coli” OR “E. coli” OR “Escherichia coli”) | Jan 10, 2024 | Jan 10, 2024 | 0 | 0 |  |
| 0(aliment OR "maladies d’origine  hydrique" OR "zoonotiques" OR cas OR maladie OR contaminent OR éclosion OR rappel OR toxique OR avis OR alerte OR malade) AND (pseudomonas OU botulisme) | Jan 10, 2024 | Jan 10, 2024 | 0 | 0 |  |

**Media outlet 3: National Post**

|  | Reviewer 1 (HS) | Reviewer 2 (VP) | Reviewer 1 (HS) | Reviewer 2 (VP) |  |
| --- | --- | --- | --- | --- | --- |
| **Search String** | **Date of Search** | **Date of Search** | **Number of Results** | **Number of Results** | **Links to Articles** |
| (food OR waterborne OR zoono* OR case? OR illness* OR contamina* OR outbreak? OR recall* OR toxic* OR advisor* OR alert* OR sick* OR disease*) AND (norovirus OR salmonella OR cyclospora OR hepatitis OR hep OR listeria OR listeriosis  OR vibrio OR Ecoli OR "E.coli" OR “E coli” OR “E. coli” OR “Escherichia coli” OR Pseudomonas OR botulism) | Jan 3, 2024 | Jan 11, 2024 | 82 | 44 | <https://nationalpost.com/news/canada/organic-strawberries-linked-to-hepatitis-a-outbreaks-in-u-s-and-canada>  <https://nationalpost.com/news/canada/chicken-nuggets-linked-to-salmonella-outbreak> |
| (aliment OR "maladies d’origine hydrique" OR "zoonotiques" OR cas OR maladie OR contaminent OR éclosion OR rappel OR toxique OR avis OR alerte OR malade) AND (norovirus OR salmonella OR Cyclospora) | Jan 10, 2024 | Jan 10, 2024 | 0 | 0 |  |
| (aliment OR "maladies d’origine hydrique" OR "zoonotiques" OR cas OR maladie OR contaminent OR éclosion OR rappel OR toxique OR avis OR alerte OR malade) AND (hépatite OR listeria OR listériose) | Jan 10, 2024 | Jan 10, 2024 | 0 | 0 |  |
| (aliment OR "maladies d’origine  hydrique" OR "zoonotiques" OR cas OR maladie OR contaminent OR éclosion OR rappel OR toxique OR avis OR alerte OR malade) AND (vibrio OR Ecoli OR “E.coli”) | Jan 10, 2024 | Jan 10, 2024 | 0 | 0 |  |
| (aliment OR "maladies d’origine  hydrique" OR "zoonotiques" OR cas OR maladie OR contaminent OR éclosion OR rappel OR toxique OR avis OR alerte OR malade) AND (“E coli” OR “E. coli” OR “Escherichia coli”) | Jan 10, 2024 | Jan 10, 2024 | 0 | 0 |  |
| (aliment OR "maladies d’origine  hydrique" OR "zoonotiques" OR cas OR maladie OR contaminent OR éclosion OR rappel OR toxique OR avis OR alerte OR malade) AND (pseudomonas OU botulisme) | Jan 10, 2024 | Jan 10, 2024 | 0 | 0 |  |

**Media outlet 4: Ottawa Citizen**

|  | Reviewer 1 (HS) | Reviewer 2 (VP) | Reviewer 1 (HS) | Reviewer 2 (VP) |  |
| --- | --- | --- | --- | --- | --- |
| **Search String** | **Date of Search** | **Date of Search** | **Number of Results** | **Number of Results** | **Links to Articles** |
| (food OR waterborne OR zoono* OR case? OR illness* OR contamina* OR outbreak? OR recall* OR toxic* OR advisor* OR alert* OR sick* OR disease*) AND (norovirus OR salmonella OR cyclospora OR hepatitis OR hep OR listeria OR listeriosis  OR vibrio OR Ecoli OR "E.coli" OR “E coli” OR “E. coli” OR “Escherichia coli” OR Pseudomonas OR botulism) | Jan 3, 2024 | Jan 11, 2024 | 43 | 17 | <https://ottawacitizen.com/news/local-news/chicken-burgers-sold-at-loblaw-stores-recalled-due-to-possible-salmonella-contamination>  <https://ottawacitizen.com/news/local-news/throw-out-sunflower-crisp-salad-kits-health-officials-warn-as-16-sickened-by-e-coli>  <https://ottawacitizen.com/news/national/federal-agency-expands-recall-of-flour-for-suspected-contamination>  <https://ottawacitizen.com/news/local-news/flour-recall-for-e-coli-risk-updated-again-with-new-brands> |
| (aliment OR "maladies d’origine hydrique" OR "zoonotiques" OR cas OR maladie OR contaminent OR éclosion OR rappel OR toxique OR avis OR alerte OR malade) AND (norovirus OR salmonella OR Cyclospora) | Jan 10, 2024 | Jan 10, 2024 | 0 | 0 |  |
| (aliment OR "maladies d’origine hydrique" OR "zoonotiques" OR cas OR maladie OR contaminent OR éclosion OR rappel OR toxique OR avis OR alerte OR malade) AND (hépatite OU listeria OU listériose) | Jan 10, 2024 | Jan 10, 2024 | 0 | 0 |  |
| (aliment OR "maladies d’origine  hydrique" OR "zoonotiques" OR cas OR maladie OR contaminent OR éclosion OR rappel OR toxique OR avis OR alerte OR malade) AND (vibrio OR Ecoli OR “E.coli”) | Jan 10, 2024 | Jan 10, 2024 | 0 | 0 |  |
| (aliment OR "maladies d’origine  hydrique" OR "zoonotiques" OR cas OR maladie OR contaminent OR éclosion OR rappel OR toxique OR avis OR alerte OR malade) AND (“E coli” OR “E. coli” OR “Escherichia coli”) | Jan 10, 2024 | Jan 10, 2024 | 0 | 0 |  |
| 0(aliment OR "maladies d’origine  hydrique" OR "zoonotiques" OR cas OR maladie OR contaminent OR éclosion OR rappel OR toxique OR avis OR alerte OR malade) AND (pseudomonas OU botulisme) | Jan 10, 2024 | Jan 10, 2024 | 0 | 0 |  |
