# Supplementary material for: Assessing the Dissemination of Federal Risk Communication by News Media Outlets During Enteric Illness Outbreaks: Canadian Content Analysis
Source: JMIR Public Health Surveill. 2025 Apr 10;11:e68724. doi: 10.2196/68724 (PMC12005601; doi:10.2196/68724)
Supplement: Multimedia Appendix 2 [file publichealth-v11-e68724-s002.docx]

Table 1: Multi-jurisdictional enteric illness outbreaks in Canada from 2014 to 2023

| **Year** | **Source of Multi-Jurisdictional Outbreak** | **Food Recall (Y/N)** | **Corresponding PHN** | **Existing Social Media Post (Y/N)** | **Number of Detected Cases** | **Number of Detected and Hospitalized Cases** | **Number of Detected and Fatal Cases*** |
| --- | --- | --- | --- | --- | --- | --- | --- |
| 2014 | Snakes and Rodents | N | PHN1 |  | 22 | 3 | 0 |
|  | Bearded Dragon | N | PHN2 |  | 4 | 2 | 0 |
|  | Chia seed powder | Y | PHN3 |  | 63 | 12 | 0 |
| 2015 | Caramel Apples | Y | PHN4 |  | 1 | Unknown | Unknown |
|  | Leafy Greens | N | PHN5 |  | 13 | Unknown | Unknown |
|  | Live Baby Poultry | N | PHN6 |  | 61 | 9 | Unknown |
|  | Frozen Breaded Chicken Products | Y | PHN7 |  | 51 | 12 | 0 |
|  | E.coli Outbreak | N | PHN8 |  | 29 | 7 | Unknown |
|  | Cyclospora Outbreak | N | PHN9 |  | 97 | 2 | 0 |
|  | Raw Shellfish | Y | PHN10 |  | 82 | 1 | 0 |
| 2016 | Salmonella Outbreaks | N | PHN11 |  | 110 | 21 | 0 |
|  | Packaged Salad from Dole Processing Facility | Y | PHN12 |  | 14 | 14 | 3 |
|  | Organic Berry Cherry Blend Frozen Fruit | Y | PHN13 |  | 25 | 10 | Unknown |
| 2017 | Raw and Undercooked Oysters | N | PHN14 |  | 331 | Unknown | 0 |
|  | Flour and Flour Products | Y | PHN15 |  | 30 | 8 | 0 |
|  | Cyclospora Outbreak | N | PHN16 |  | 164 | Unknown | Unknown |
| 2018 | Romaine Lettuce | N | PHN17 |  | 42 | 17 | 1 |
|  | Romaine Lettuce | N | PHN18 |  | 8 | 1 | 0 |
|  | Raw Oysters | N | PHN19 |  | 176 | Unknown | 0 |
|  | Long English Cucumbers | N | PHN20 |  | 56 | 11 | 0 |
| 2019 | Romaine Lettuce | N | PHN21 |  | 29 | 10 | 0 |
|  | Raw Chicken, Frozen Breaded Chicken Products | Y | PHN22 |  | 584 | 97 | 3 |
|  | Celebrate Cream Puffs and Mini Chocolate Eclairs | Y | PHN23 |  | 85 | 22 | 3 |
|  | Rosemount Cooked Diced Chicken | Y | PHN24 |  | 7 | 6 | Unknown |
|  | Filicetti Dry, Cured Sausages | Y | PHN25 |  | 13 | 1 | 0 |
| 2020 | Romaine Lettuce | N | PHN26 |  | 4 | 1 | 0 |
|  | Fresh Express Salad Kits | Y | PHN27 |  | 28 | 8 | 0 |
|  | Raw Turkey and Raw Chicken | N | PHN28 |  | 130 | 39 | 1 |
|  | Carnivora Frozen Raw Pet Food | Y | PHN29 |  | 5 | 2 | 0 |
|  | Red Onions | Y | PHN30 |  | 515 | 79 | 3 |
|  | Peaches | Y | PHN31 |  | 57 | 12 | 0 |
|  | Salad Products | Y | PHN32 |  | 370 | 10 | 0 |
|  | Shellfish | N | PHN33 |  | 23 | 1 | 0 |
|  | Pig Ear Dog Treats | N | PHN34 |  | 10 | 3 | 1 |
|  | Pet Hedgehogs | N | PHN35 |  | 31 | 4 | 0 |
| 2021 | Snakes and Rodents | N | PHN36 |  | 106 | 7 | 0 |
|  | Eggs | Y | PHN37 |  | 70 | 19 | 0 |
|  | Frozen Mangoes | Y | PHN38 |  | 3 | 2 | 0 |
| 2022 | Frozen Whole Corn | Y | PHN39 |  | 118 | 4 | 2 |
|  | Hankook Kimchi | Y | PHN40 |  | 14 | 0 | 0 |
|  | Raw Oysters | Y | PHN41 |  | 339 | Unknown | 0 |
|  | Spot Prawns | Y | PHN42 |  | 60 | Unknown | 0 |
|  | Organic Strawberries | N | PHN43 |  | 10 | 4 | 0 |
| 2023 | Snakes and Rodents** | N | PHN44 |  | 76 | 10 | 1 |
|  | Sardines from Bordeaux, France | N | PHN45 |  | 15 | Unknown | 1 |
|  | Raw pet food and contact with cattle** | N | PHN46 |  | 44 | 13 | 0 |

*Cause of death was not necessarily related to the enteric pathogen

**Ongoing outbreaks at the time of data collection
