# Supplementary material for: Assessing the Dissemination of Federal Risk Communication by News Media Outlets During Enteric Illness Outbreaks: Canadian Content Analysis
Source: JMIR Public Health Surveill. 2025 Apr 10;11:e68724. doi: 10.2196/68724 (PMC12005601; doi:10.2196/68724)
Supplement: Multimedia Appendix 3 [file publichealth-v11-e68724-s003.docx]

Supplementary File 3: Codebook Used for Media Content Analysis

| **Variable** | **Definition** | **Example** | **Code** |
| --- | --- | --- | --- |
| **General** | | | |
| UNIQUE_ID | Indicate the unique ID assigned to this article  *Following de-duplication, a unique ID was assigned to each article. On the excel sheet containing data to be analyzed, IDs are denoted as ART*** to indicate “article” and its corresponding number*  **** the specific number assigned to the URL. IDs range from MED001 to (MED763)* |  | [Text entry] |
| DATE | Indicate the year when the article was posted |  | 1. Number entry [YYYY] |
| GEOGRAPHICAL_SCOPE | Indicate the level of coverage provided by the media outlet | ***Local*** *media provides information the community or city level*  ***Regional*** *media cover information pertaining to a province or territory*  ***National*** *media provides coverage for the entire country* | 1. Local 2. Regional 3. National |
| MODIFICATION | Indicate whether this communication product replicates the PHN verbatim  ***If 1, 3, 4, or 5 is chosen, code as 99.8, where applicable.*** |  | 1. Yes, it replicates the PHN verbatim 2. No, it does not replicate the PHN or other communication products 3. Yes, it replicates CFIA’s food recall notices verbatim 4. Yes, replicates other Canada.ca communication verbatim (e.g., statement by the Canadian Medical Officer of Health) 5. Yes, it replicates official industry communication verbatim (e.g., Safety Notice by Producer) |
| LANGUAGE | Indicate whether this article is written in English or French |  | 1. English 2. French   99.8 – Not modified |
| INTENTION_OF_NOTICE | Indicate whether this article alerts audience about an ongoing outbreak, provides an update to an ongoing outbreak, notifies audience of an outbreak that has concluded, notifies audience of a food recall, or provides information about adhering to best practices  **Duplicate will be assigned to articles that contain the same content as another article but is issued by a different publisher* |  | 1. Notice of Outbreak 2. Update 3. End of Outbreak or Advisory 4. Food Recall Notice 5. Reminder or Best Practices 6. Duplicate 7. Unclear 8. Other   99.8 – Not modified |
| **Content-Specific Variables** | | | |
| **Outbreak Characteristics** | | | |
| SOURCE | Indicate whether the source of the outbreak is foodborne or zoonotic  *To be coded as “unknown”, the article must clearly state that the source of the outbreak is not known.* |  | 1. Foodborne 2. Zoonotic 3. Unknown 4. Other 5. Does not mention   99.8 – Not modified |
| RECALL | Indicate whether the article makes mention of the source of outbreak being recalled  *Code as 99.9 if the outbreak was not associated with a recall or if a recall was not in effect at the time of publication* |  | 1. Yes 2. No   99.8 – Not modified  99.9 – N/A |
| LOCATION_OF_OUTBREAK | Which of the following provinces/territories were involved in the outbreak, as specified in the article  *(Select all that apply)* |  | 1. Alberta 2. British Columbia 3. Manitoba 4. New Brunswick 5. Newfoundland and Labrador 6. Northwest Territories 7. Nova Scotia 8. Nunavut 9. Ontario 10. Prince Edward Island 11. Quebec 12. Saskatchewan 13. Yukon 14. National (across Canada) 15. International 16. Does not mention   99.8 – Not modified |
| **Investigation Summary** | | | |
| CASES | Indicate if the number of cases is directly mentioned in the article  *Number of cases refer to the number of individuals infected because of the outbreak. This value includes laboratory-confirmed and probably primary cases*  *Code as 99.9 is the PHN does not indicate the number of illnesses* |  | 1. Yes 2. No   99.8 – Not modified  99.9 – N/A |
| HOSPITALIZATIONS | Indicate if the number of hospitalizations is directly mentioned in the article  *Code as 99.9 is the PHN does not indicate the number of hospitalizations* |  | 1. Yes 2. No   99.8 – Not modified  99.9 – N/A |
| DEATHS | Indicate if the number of deaths is directly mentioned in the article  *Code as 99.9 is the PHN does not indicate the number of deaths* |  | 1. Yes 2. No   99.8 – Not modified  99.9 – N/A |
| **What you Should do to Protect your Health** | | | |
| BEHAVIOUR | Indicate whether the article clearly describes how to protect oneself from the undesirable outcome in detail | *Example (#1): Wash your hands with soap and warm water for at least 20 seconds immediately following contact with any of the recalled products (with a link to a recalled products)*  *Example (#2): It is critical individuals use safe and effective handwashing practices* | 1. Yes, the article **provides specific behavioural recommendations** 2. No, the article **does not provide behavioral recommendations**   99.8 – Not modified |
| BEHAVIOUR_B | Indicate whether the article contains information on steps to take if you become ill |  | 1. Yes 2. No   99.8 – Not modified 99.9 – N/A |
| **Symptoms** | | | |
| SYMPTOMS | Indicate whether the article indicates symptoms that can occur in those who become ill |  | 1. Yes 2. No   99.8 – Not modified |
| **Additional Information and Investigation History** | | | |
| **Public Inquiries** | | | |
| PUBLIC_INQ | Indicate whether the article contains information that the public can use for inquiries | *Example: toll-free number, email* | 1. Yes 2. No   99.8 – Not modified |
| **Health Belief Model (HBM)** | | | |
| CUE_TO_ACTION | Indicate whether the article contains a cue to action.  *Cues to action are stimuli that promote the implementation of recommended behaviours.* | *“If you think you became sick from consuming a recalled product, call your local health centre (with a link to recalled to products)”* | 1. Yes, the article contains one or more cues to action 2. No, the article does not contain any cues to action   99.8 – Not modified  99.9 – N/A |
| SELF_EFFICACY | Indicate whether the article contains information that would increase an individual’s confidence in implementing the recommended behaviours | *For example, the article may clearly lay out how to disinfect surfaces prior to handling chicken* | 1. Yes, the article contains direct information about how successful the recommendations are in preventing illness 2. No, the article does not contain direct information about how successful the recommendations are in preventing illness   99.8 – Not modified |
| SUSCEPTIBILITY | Indicate whether the article provides direct information about the individual’s vulnerability to the undesirable outcome | *Examples can include mentioning the number of deaths, mentioning that the pathogen is easily spread, or mentioning specific characteristics one might possess that make them more susceptible (e.g., pregnant women are at an increased risk for illness)* | 1. Yes, the article provides direct information about the individual’s vulnerability to the undesirable outcome 2. No, the article does not provide direct information about the individual’s vulnerability to the undesirable outcome   99.8 – Not modified  99.9 – N/A |
| SEVERITY | Indicate whether the article provides direct information about the seriousness and consequences of the outbreak (e.g., becoming severely ill) | *Examples can include mentioning the number of deaths and hospitalizations* | 1. Yes, the article provides direct information about the severity to the undesirable outcome 2. No, the article does not provide direct information about the severity to the undesirable outcome   99.8 – Not modified  99.9 – N/A |
| BENEFITS | Indicate whether this article highlights the benefits of implementing the recommended behavior(s) | *“Implementing the following behaviors can reduce the risk of illness”* | 1. Yes, the article highlights benefits of implementing recommended behaviors 2. No, the article does not highlight benefits of implementing recommended behaviors   99.8 – Not modified  99.9 – N/A |
| BARRIERS | Indicate whether this article addresses and provides directions to overcome obstacles, or factors that may **hinder** a person from performing the recommended behavior(s) | *A general example includes a article addressing perceived ineffectiveness, or the belief that the recommend behaviour is ineffective.* | 1. Yes, the article addresses and provides information to overcome barriers hindering an individual from performing the recommended behaviour 2. No, the article does not address nor provide information to overcome barriers hindering an individual from performing the recommended behaviour   99.8 – Not modified  99.9 – N/A |
