# Supplementary material for: Assessing the Dissemination of Federal Risk Communication by News Media Outlets During Enteric Illness Outbreaks: Canadian Content Analysis
Source: JMIR Public Health Surveill. 2025 Apr 10;11:e68724. doi: 10.2196/68724 (PMC12005601; doi:10.2196/68724)
Supplement: Multimedia Appendix 4 [file publichealth-v11-e68724-s004.docx]

Table 1: Distribution of multi-jurisdictional outbreaks by number of media articles issued

| Multi-Jurisdictional Outbreak | Number of Corresponding Media Articles |
| --- | --- |
| 1. Contact with snakes and rodents (2014) 2. Contact with bearded dragons (2014) 3. Leafy greens (2015) 4. E. coli outbreak (2015) 5. Raw shellfish (2015) 6. Raw and undercooked oysters (2017) 7. Cyclospora outbreak (2017) 8. Romaine lettuce (2018) 9. Long English cucumbers (2018) 10. Romaine lettuce (2020) 11. Raw turkey and chicken (2020) 12. Carnivora frozen raw pet food 13. Peaches (2020) 14. Salad products (2020) 15. Shellfish (2020) 16. Pig ear dog treats (2020) 17. Pet hedgehogs (2020) 18. Contact with snakes and rodents (2021) 19. Eggs (2020) 20. Hankook Kimchi (2022) 21. Spot prawns (2022) 22. Organic strawberries (2022) 23. Contact with snakes and rodents (2023) 24. Sardines from Bordeaux, France (2023) 25. Raw pet food and contact with cattle (2023) | 0 – 10 |
| 1. Chia seed powder (2014) 2. Frozen breaded chicken products (2015) 3. Cyclospora outbreak (2015) 4. Salmonella outbreak (2016) 5. Dole processing facility (2016) 6. Raw oysters (2018) 7. Celebrate cream puffs and mini chocolate eclairs (2019) 8. Rosemount cooked diced chicken (2019) 9. Filicetti dry, cured sausages (2019) 10. Fresh Express salad kits (2020) 11. Frozen whole corn (2022) | 11 - 21 |
| 1. Caramel apples (2015) 2. Romaine lettuce (2018) 3. Frozen mangoes (2021) 4. Raw oysters (2022) | 22 - 32 |
| 1. Organic Berry Cherry Blend frozen fruit 2. Romaine lettuce (2019) | 33 - 43 |
| 1. Red onions (2020) | 44 - 54 |
| 1. Contact with live baby poultry | 55 - 65 |
|  | 66 - 76 |
|  | 77 - 87 |
| 1. Flour and flour products (2017) | 88 - 98 |
| 1. Raw chicken and frozen raw breaded chicken products (2017 – 2019) | 99 - 109 |
