# Supplementary material for: Assessing the Dissemination of Federal Risk Communication by News Media Outlets During Enteric Illness Outbreaks: Canadian Content Analysis
Source: JMIR Public Health Surveill. 2025 Apr 10;11:e68724. doi: 10.2196/68724 (PMC12005601; doi:10.2196/68724)
Supplement: Multimedia Appendix 5 [file publichealth-v11-e68724-s005.docx]

**Table 1**: Descriptive statistics of communication variables stratified by scope of news media coverage

|  |  |  |  |  |  |  |  |
| --- | --- | --- | --- | --- | --- | --- | --- |
|  |  |  | **Overall** | **Local** | **Regional** | **National** | **Chi-Square test** |
| **Category** | **Variable** | **Sub-Variable** | **n (%)** | **n (%)** | **n (%)** | **n (%)** | **X^2^ (df)**  **p-value** |
| General Characteristics of Media Communication Products | Geographical Scope of Media Outlets | Regional | 123 (38.4) | N/A | 123 (100) | N/A | N/A |
|  |  | Local | 100 (31.3) | 100 (100) | N/A | N/A |  |
|  |  | National | 97 (30.3) | N/A | N/A | 97 (100) |  |
|  |  | Total | 320 | 100 | 123 | 97 |  |
|  | Language of Communication Products | English | 533 (96.0) | 238 (99.6) | 188 (100) | 107 (83.6) | 67.7 (2)  **<0.001** |
|  |  | French | 22 (4.0) | 1 (0.4) | 0 | 21 (16.4) |  |
|  |  | Total | 555 | 239 | 188 | 128 |  |
|  | Intention of Communication Product | Notice of Outbreak or Advisory | 107 (33.4) | 30 (30.0) | 41 (33.3) | 36 (37.1) | 20.0 (12)  0.07 |
|  |  | Update of Ongoing Outbreak | 30 (9.4) | 7 (7.0) | 15 (12.2) | 8 (8.2) |  |
|  |  | End of Outbreak or Advisory | 12 (3.8) | 1 (1.0) | 3 (2.4) | 8 (8.2) |  |
|  |  | Food Recall Notice | 121 (37.8) | 44 (44.0) | 44 (35.8) | 33 (34.0) |  |
|  |  | Reminder or Best Practice | 19 (5.9) | 10 (10.0) | 4 (3.3) | 5 (5.2) |  |
|  |  | Unclear | 30 (9.4) | 8 (8.0) | 15 (12.2) | 7 (7.2) |  |
|  |  | Other | 1 (0.3) | 0 | 1 (0.8) | 0 |  |
|  |  | Total | 320 | 100 | 123 | 97 |  |
|  | Source of Outbreak | Foodborne | 286 (89.3) | 87 (87.0) | 111 (90.2) | 88 (90.7) | 2.9 (4)  0.6 |
|  |  | Zoonotic | 7 (2.2) | 4 (4.0) | 1 (0.8) | 2 (2.1) |  |
|  |  | Unknown | 27 (100) | 9 (9.0) | 11 (8.9) | 7 (7.2) |  |
|  |  | Total | 320 | 100 | 123 | 97 |  |
|  | Indication of Item Recall | Yes | 194 (95.1) | 68 (94.4) | 74 (94.9) | 52 (96.3) | 0.2 (2)  0.9 |
|  |  | No | 10 (4.9) | 4 (5.6) | 4 (5.1) | 2 (3.7) |  |
|  |  | Total Applicable | 204 | 72 | 78 | 54 |  |
|  |  | N/A | 116 | 28 | 45 | 43 |  |
|  |  | Total | 320 | 100 | 123 | 97 |  |
|  | Indication of Outbreak Location | Yes | 181 (57.3) | 48 (48.0) | 72 (58.5) | 61 (65.6) | 6.2 (2)  **0.04** |
|  |  | No | 135 (42.7) | 52 (52.0) | 51 (41.5) | 32 (34.4) |  |
|  |  | Total Applicable | 316 | 100 | 123 | 93 |  |
|  |  | N/A | 4 | 0 | 0 | 4 |  |
|  |  | Total | 320 | 100 | 123 | 97 |  |
|  | Indication of the Number of Cases | Yes | 237 (74.3) | 66 (66.0) | 97 (78.9) | 74 (77.1) | 5.3 (2)  0.07 |
|  |  | No | 82 (25.7) | 34 (34.0) | 26 (21.1) | 22 (22.9) |  |
|  |  | Total Applicable | 319 | 100 | 123 | 96 |  |
|  |  | N/A | 1 | 0 | 0 | 1 |  |
|  |  | Total | 320 | 100 | 123 | 97 |  |
|  | Indication of the Number of Hospitalizations | Yes | 144 (52.0) | 33 (35.1) | 63 (61.2) | 48 (60.0) | 16.3 (2)  **<0.001** |
|  |  | No | 133 (48.0) | 61 (64.9) | 40 (38.8) | 32 (40.0) |  |
|  |  | Total Applicable | 277 | 94 | 103 | 80 |  |
|  |  | N/A | 43 | 6 | 20 | 17 |  |
|  |  | Total | 320 | 100 | 123 | 97 |  |
|  | Indication of the Number of Deaths | Yes | 114 (43.8) | 24 (31.2) | 50 (49.0) | 40 (49.4) | 7.1 (2)  **0.03** |
|  |  | No | 146 (56.2) | 53 (68.8) | 52 (51.0) | 41 (50.6) |  |
|  |  | Total Applicable | 260 | 77 | 102 | 81 |  |
|  |  | N/A | 60 | 23 | 21 | 16 |  |
|  |  | Total | 320 | 100 | 123 | 97 |  |
|  | Provision of Behavioural Recommendations | Yes | 243 (75.9) | 81 (81.0) | 94 (76.4) | 68 (70.1) | 3.2 (2)  0.2 |
|  |  | No | 77 (24.1) | 19 (19.0) | 29 (23.6) | 29 (29.9) |  |
|  |  | Total Applicable | 320 | 100 | 123 | 97 |  |
|  |  | N/A | 0 | 0 | 0 | 0 |  |
|  |  | Total | 320 | 100 | 123 | 97 |  |
|  | Provision of Behavioural Recommendations if Individual Becomes Ill | Yes | 62 (25.5) | 29 (36.3) | 24 (25.5) | 9 (13.2) | 9.9 (2)  **0.01** |
|  |  | No | 181 (74.5) | 52 (63.7) | 70 (74.5) | 59 (86.8) |  |
|  |  | Total Applicable | 243 | 81 | 94 | 68 |  |
|  |  | N/A | 77 | 19 | 29 | 29 |  |
|  |  | Total | 320 | 100 | 123 | 97 |  |
|  | Indication of Symptoms | Yes | 201 (62.8) | 65 (65.0) | 75 (61.0) | 61 (62.9) | 0.4 (2)  0.8 |
|  |  | No | 119 (37.2) | 35 (35.0) | 48 (39.0) | 36 (37.1) |  |
|  |  | Total | 320 | 100 | 123 | 97 |  |
| Presence of the Health Belief Model Constructs | Presence of Cue to Action | Yes | 202 (83.1) | 70 (86.4) | 80 (85.1) | 52 (76.4) | 3.0 (2)  0.2 |
|  |  | No | 41 (16.9) | 11 (13.6) | 14 (14.9) | 16 (23.5) |  |
|  |  | Total | 243 | 81 | 94 | 68 |  |
|  | Presence of Self-Efficacy | Yes | 191 (78.6) | 68 (84.0) | 79 (84.0) | 44 (64.7) | 10.8 (2)  **<0.01** |
|  |  | No | 52 (21.4) | 13 (16.0) | 15 (16.0) | 24 (35.3) |  |
|  |  | Total | 243 | 81 | 94 | 68 |  |
|  | Presence of Susceptibility | Yes | 190 (78.2) | 62 (76.5) | 78 (83.0) | 50 (73.5) | 2.3 (2)  0.3 |
|  |  | No | 53 (21.8) | 19 (23.5) | 16 (17.0) | 18 (26.5) |  |
|  |  | Total | 243 | 81 | 94 | 68 |  |
|  | Presence of Severity | Yes | 161 (66.3) | 54 (66.7) | 67 (71.3) | 40 (58.8) | 2.7 (2)  0.3 |
|  |  | No | 82 (33.7) | 27 (33.3) | 27 (28.7) | 28 (41.2) |  |
|  |  | Total | 243 | 81 | 94 | 68 |  |
|  | Presence of Benefit | Yes | 50 (20.6) | 17 (21.0) | 23 (24.5) | 10 (14.7) | 2.3 (2)  0.3 |
|  |  | No | 193 (79.4) | 64 (79.0) | 71 (75.5) | 58 (85.3) |  |
|  |  | Total | 243 | 81 | 94 | 68 |  |
|  | Presence of Barrier | Yes | 15 (6.2) | 6 (7.4) | 7 (7.4) | 2 (2.9) | 1.7 (2)  0.4 |
|  |  | No | 228 (93.8) | 75 (92.6) | 87 (92.6) | 66 (97.1) |  |
|  |  | Total | 243 | 81 | 94 | 68 |  |

**Table 2**: Descriptive statistics of communication variables stratified by source of outbreak

|  |  |  |  |  |  | |  |  |
| --- | --- | --- | --- | --- | --- | --- | --- | --- |
|  |  |  | **Overall** | **Foodborne** | | **Zoonotic** | **Unknown** | **Chi-Square Test** |
| **Category** | **Variable** | **Sub-Variable** | **n (%)** | **n (%)** | **n (%)** | | **n(%)** | **X^2^ (df)**  **p-value** |
| General Characteristics of Media Communication Products | Geographical Scope of Media Outlets | Local | 100 (31.3) | 87 (30.4) | 4 (57.2) | | 9 (33,3) | 1.4 (2)  0.5 |
|  |  | Regional | 123 (38.4) | 111 (38.8) | 1 (14.2) | | 11 (40.7) |  |
|  |  | National | 97 (30.3) | 88 (30.8) | 2 (28.6) | | 7 (25.9) |  |
|  |  | Total | 320 | 286 | 7 | | 27 |  |
|  | Language of Modified Communication Products | English | 533 (96.0) | 440 (95.6) | 62 (100) | | 31 (93.9) | 1.7 (1)  0.2 |
|  |  | French | 22 (4.0) | 20 (4.4) | 0 | | 2 (6.1) |  |
|  |  | Total | 555 | 460 | 62 | | 33 |  |
|  | Intention of Communication Product | Notice of Outbreak or Advisory | 107 (33.4) | 81 (28.3) | 5 (71.4) | | 21 (77.8) | 8.5 (6)  0.2 |
|  |  | Update of Ongoing Outbreak | 30 (9.4) | 29 (10.1) | 1 (14.3) | | 0 |  |
|  |  | End of Outbreak or Advisory | 12 (3.8) | 12 (4.2) | 0 | | 0 |  |
|  |  | Food Recall Notice | 121 (37.8) | 121 (42.3) | 0 | | 0 |  |
|  |  | Reminder or Best Practice | 19 (5.9) | 19 (6.6) | 0 | | 0 |  |
|  |  | Unclear | 30 (9.4) | 23 (8.0) | 1 (14.3) | | 6 (22.2) |  |
|  |  | Other | 1 (0.3) | 1 (0.3) | 0 | | 0 |  |
|  |  | Total | 320 | 286 | 7 | | 27 |  |
|  | Source of Outbreak | Foodborne | 286 (89.3) | 286 (100) | N/A | | N/A | N/A |
|  |  | Zoonotic | 7 (2.2) | N/A | 7 (100) | | N/A |  |
|  |  | Unknown | 27 (100) | N/A | N/A | | 27 (100) |  |
|  |  | Total | 320 | 286 | 7 | | 27 |  |
|  | Indication of Item Recall | Yes | 194 (95.1) | 194 (95.1) | N/A | | N/A | N/A |
|  |  | No | 10 (4.9) | 10 (4.9) | N/A | | N/A |  |
|  |  | Total Applicable | 204 | 204 | N/A | | N/A |  |
|  |  | N/A | 116 | 82 | 7 | | 27 |  |
|  |  | Total | 320 | 286 | 7 | | 27 |  |
|  | Indication of Outbreak Location | Yes | 181 (57.3) | 152 (53.5) | 6 (85.7) | | 23 (92.0) | 1.7 (1)  0.2 |
|  |  | No | 135 (42.7) | 132 (46.5) | 1 (14.3) | | 2 (7.0) |  |
|  |  | Total Applicable | 316 | 284 | 7 | | 25 |  |
|  |  | N/A | 4 | 2 | 0 | | 2 |  |
|  |  | Total | 320 | 286 | 7 | | 27 |  |
|  | Indication of the Number of Cases | Yes | 237 (74.3) | 203 (71.2) | 7 (100) | | 27 (100) | 1.6 (1)  0.2 |
|  |  | No | 82 (25.7) | 82 (28.8) | 0 | | 0 |  |
|  |  | Total Applicable | 319 | 285 | 7 | | 27 |  |
|  |  | N/A | 1 | 1 | 0 | | 0 |  |
|  |  | Total | 320 | 286 | 7 | | 27 |  |
|  | Indication of the Number of Hospitalization | Yes | 144 (52.0) | 120 (49.2) | 4 (57.1) | | 20 (76.9) | 0.001 (1)  0.9 |
|  |  | No | 133 (48.0) | 124 (50.8) | 3 (42.9) | | 6 (23.1) |  |
|  |  | Total Applicable | 277 | 244 | 7 | | 26 |  |
|  |  | N/A | 143 | 142 | 0 | | 1 |  |
|  |  | Total | 320 | 286 | 7 | | 27 |  |
|  | Indication of the Number of Deaths | Yes | 114 (43.8) | 99 (42.1) | 2 (100) | | 13 (56.2) | 0.9 (1)  0.4 |
|  |  | No | 146 (56.2) | 136 (57.9) | 0 | | 10 (43.5) |  |
|  |  | Total Applicable | 260 | 235 | 2 | | 23 |  |
|  |  | N/A | 60 | 51 | 5 | | 4 |  |
|  |  | Total | 320 | 286 | 7 | | 27 |  |
|  | Provision of Behavioural Recommendations to Prevent Illness | Yes | 243 (75.9) | 228 (79.7) | 4 (57.1) | | 11(40.7) | 1.0 (1)  0.3 |
|  |  | No | 77 (24.1) | 58 (20.3) | 3 (42.9) | | 16 (59.3) |  |
|  |  | Total | 320 | 286 | 7 | | 27 |  |
|  | Provision of Behavioural Recommendations to if Individual Becomes Ill | Yes | 62 (25.5) | 62 (27.2) | 0 | | 0 | 8.5 (1)  **<0.01** |
|  |  | No | 181 (74.5) | 166 (72.8) | 4 (100) | | 11 (100) |  |
|  |  | Total Applicable | 243 | 228 | 4 | | 11 |  |
|  |  | N/A | 77 | 58 | 3 | | 16 |  |
|  |  | Total | 320 | 286 | 7 | | 27 |  |
|  | Indication of Symptoms | Yes | 201 (62.8) | 184 (64.3) | 3 (42.9) | | 14 (51.9) | 0.6 (1)  0.4 |
|  |  | No | 119 (37.2) | 102 (35.7) | 4 (57.1) | | 13 (48.1) |  |
|  |  | Total | 320 | 286 | 7 | | 27 |  |
| Presence of the Health Belief Model Constructs | Presence of Cue to Action | Yes | 202 (83.1) | 194 (85.1) | 2 (50.0) | | 6 (54.5) | 1.5 (1)  0.2 |
|  |  | No | 41 (16.9) | 34 (14.9) | 2 (50.0) | | 5 (45.5) |  |
|  |  | Total | 243 | 228 | 4 | | 11 |  |
|  | Presence of Self-Efficacy | Yes | 191 (78.6) | 187 (82.0) | 0 | | 4 (36.4) | 12.1 (1)  **<0.001** |
|  |  | No | 52 (21.4) | 41 (18.0) | 4 (100) | | 7 (63.6) |  |
|  |  | Total | 243 | 228 | 4 | | 11 |  |
|  | Presence of Susceptibility | Yes | 190 (78.2) | 175 (76.7) | 4 (100) | | 11 (100) | 0.2 (1)  0.6 |
|  |  | No | 53 (21.8) | 53 (23.2) | 0 | | 0 |  |
|  |  | Total | 243 | 228 | 4 | | 11 |  |
|  | Presence of Severity | Yes | 161 (66.3) | 150 (65.8) | 3 (75.0) | | 8 (72.7) | 1 (1)  **<0.001** |
|  |  | No | 82 (33.7) | 78 (34.2) | 1 (25.0) | | 3 (27.3) |  |
|  |  | Total | 243 | 228 | 4 | | 11 |  |
|  | Presence of Benefit | Yes | 50 (20.6) | 43 (18.9) | 1 (25.0) | | 6 (54.5) | 1 (1)  **<0.001** |
|  |  | No | 193 (79.4) | 185 (81.1) | 3 (75.0) | | 5 (45.5) |  |
|  |  | Total | 243 | 228 | 4 | | 11 |  |
|  | Presence of Barrier | Yes | 15 (6.2) | 15 (6.6) | 0 | | 0 | 1 (1)  **<0.001** |
|  |  | No | 228 (93.8) | 213 (93.4) | 4 (100) | | 11 (100) |  |
|  |  | Total | 243 | 228 | 4 | | 11 |  |
